# TSA restores hair follicle-inductive capacity of skin-derived precursors

**DOI:** 10.1038/s41598-019-39394-w

**Published:** 2019-02-27

**Authors:** Ling Guo, Xiaoxiao Wang, Jifan Yuan, Meishu Zhu, Xiaobing Fu, Ren-He Xu, Chuanyue Wu, Yaojiong Wu

**Affiliations:** 1 State Key Laboratory of Chemical Oncogenomics, and the the Shenzhen Key Laboratory of Health Sciences and Technology, Graduate School at Shenzhen, Tsinghua University, Shenzhen, China; 2 Tsinghua-Berkeley Shenzhen Institute (TBSI), Tsinghua University, Shenzhen, China; 3 Guangdong Provincial Key Laboratory of Cell Microenvironment and Disease Research, Shenzhen Key Laboratory of Cell Microenvironment, and Department of Biology, Academy for Advanced Interdisciplinary Studies, Southern University of Science and Technology, Shenzhen, China; 4 Shenzhen Second People’s Hospital (The First Hospital Affiliated to Shenzhen University), Shenzhen, China; 5 Wound Healing and Cell Biology Laboratory, Institute of Basic Medical Science, Chinese PLA General Hospital, Beijing, China; 6 Stem Cell and Tissue Regeneration Laboratory, The First Affiliated Hospital, General Hospital of PLA, Beijing, China; 7 University of Macau, Institute of Translational Medicine, and Centre of Reproduction, Development and Aging, Faculty of Health Sciences, Taipa, Macau, China; 8 Department of Pathology, University of Pittsburgh School of Medicine, Pittsburgh, PA 15261 USA

## Abstract

The genesis of the hair follicle relies on signals derived from mesenchymal cells in the dermis during skin morphogenesis and regeneration. Multipotent skin-derived precursors (SKPs), which exhibit long term proliferation potential when being cultured in spheroids, have been shown to induce hair genesis and hair follicle regeneration in mice, implying a therapeutic potential of SKPs in hair follicle regeneration and bioengineering. However, the hair-inductive property of SKPs declines progressively upon *ex vivo* culture expansion, suggesting that the expressions of the genes responsible for hair induction are epigenetically unstable. In this study, we found that TSA markedly alleviated culture expansion induced SKP senescence, increased the expression and activity of alkaline phosphatase (AP) in the cells and importantly restored the hair inductive capacity of SKPs. TSA increased the acetylation level of histone H3, including the K19/14 sites in the promoter regions of bone morphogenetic proteins (BMPs) genes, which were associated with elevated gene expression and BMP signaling activity, suggesting a potential attribution of BMP pathway in TSA induced recovery of the hair inductive capacity of SKPs.

## Introduction

The genesis of the hair follicle relies on signals derived from mesenchymal cells in the dermis during skin morphogenesis and regeneration^1–3^. Previous studies indicate that dermal papilla (DP) cells derived from the hair follicle are able to induce hair follicle formation^4–6^. However, the application of DP cells in tissue engineering has been limited by their availability. The cells can only been isolated manually from large hair follicles in the scalp and their hair-inductive property diminishes markedly upon culture expansion^7,8^. Intriguingly, multipotent skin-derived precursors (SKPs) have recently been shown to induce hair follicle formation. SKPs express Sox2 and nestin, and exhibit long term proliferation potential when being cultured in spheroids^3,9,10^. When subcutaneously injected in mice the cells were found to incorporate into the DP and induce hair genesis^10^, and when transplanted in combination with epidermal stem cells into excisional wounds in mice, SKPs induced *de novo* hair genesis^11^. These results imply a potential application of SKPs in hair follicle regeneration and bioengineering. However, the hair-inductive property of SKPs declines progressively upon *ex vivo* culture expansion^11,12^, suggesting that the expression of the genes responsible for hair induction are epigenetically unstable.

Trichostatin A (TSA) is a potent and specific inhibitor of a histone deacetylase (HDAC) activity^13,14^. It selectively inhibits the class I and II, but not class III, mammalian HDAC families of enzymes^15^. Acetylation of K9 and K14 in histone H3 is required for the recruitment of TFIID^16^, and TFIID binding to the promoter causes DNA bending and downstream translocation of the SWI/SNF-modified nucleosome, which allows the initiation of transcription^17^. In our previous study, we have proved that the altered expression of these genes was closely associated with epigenetic dysregulation of histone H3 acetylation in K9 and K14^18^. It has been shown previously that TSA modulates a wide variety of cellular activities such as cell differentiation and proliferation depending on cell types and their functional states^14^.

In this study, we found that TSA markedly alleviated culture expansion induced SKP senescence, increased the expression and activity of AP in the cells and importantly restored the hair inductive capacity of SKPs. TSA increased the level of K19/14 acetylation in the promoter regions of bone morphogenetic proteins (*BMP*) genes, which are associated with elevated gene expression and BMP signaling activity, suggesting a potential attribution of BMP pathway in TSA induced recovery of SKPs’ hair inductive capacity.

## Results

### SKPs lose their hair follicle-inductive capacity after culture expansion

**Figure 1:**
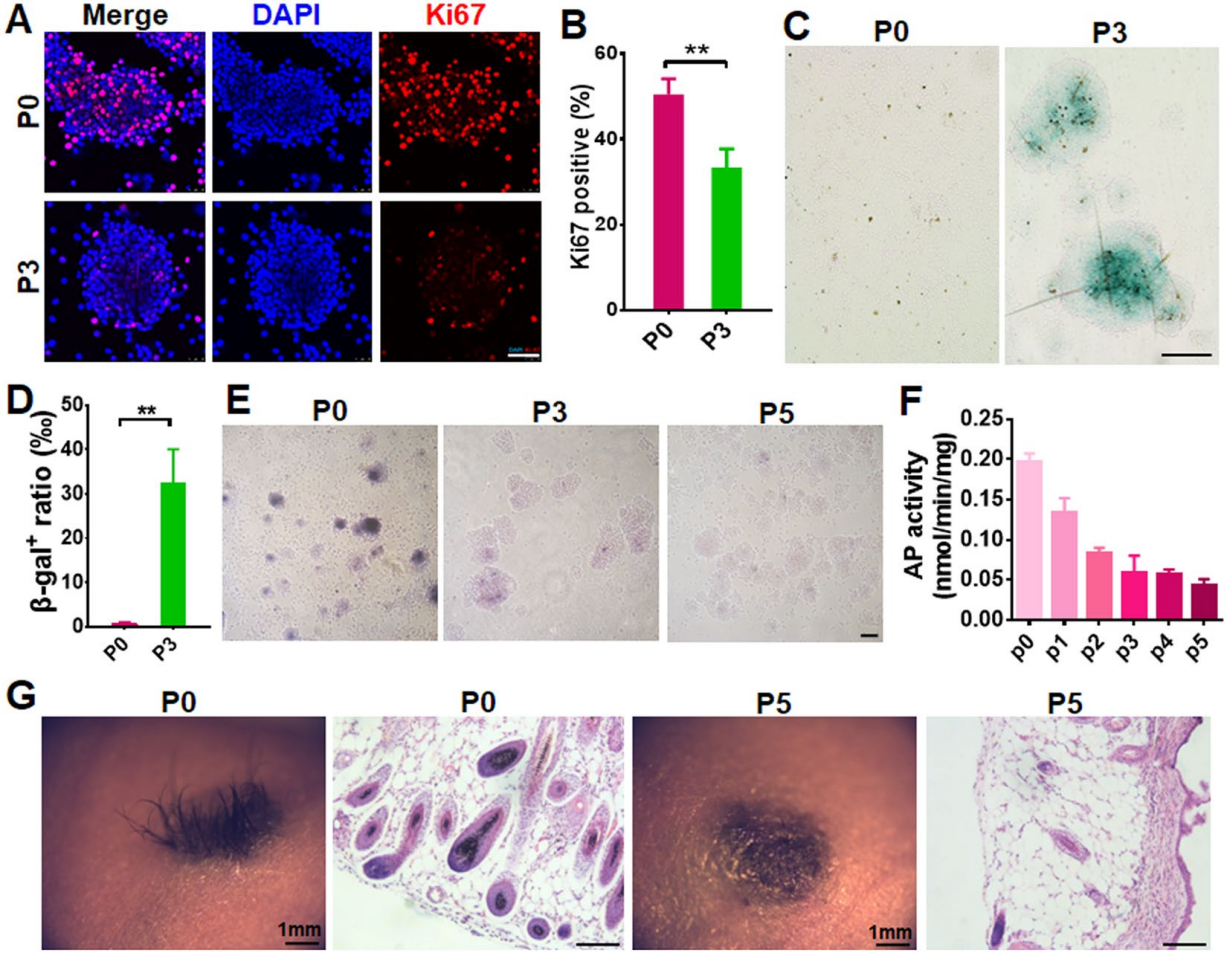
Senescence and hair induction capacity of SKPs in culture. (**A**,**B**) Passage 0 and 3 SKPs were immunostained for Ki67. Nuclei were stained with DAPI. Scale bar, 50 μm (**A**). The percentages of Ki67-positive SKPs were quantified by Image J (**B**). Data are represented as means ± SEM (n = 3; ***P < 0.001). At least 200 cells were counted in each experiment. (**C**,**D**) SKPs in different passages (P) were stained for SA-β-galactosidase (Green) for cellular senescence (C, Scale bars: 100 μm) and the percentage of cells positive for SA-β-galactosidase were counted using Image J (**D**, mean ± SEM; n = 3; ***P < 0.001) (**D**). (**E**,**F**) Representative images of SKP spheres in P0, 3 and 5 after AP stain (**E**, Scale bars: 50 μm). AP activity of SKPs in P0-5 were analyzed using an AP assay kit (**F**) (mean ± SEM; n = 3; ***P < 0.001). (**G**) Hair genesis of SKPs in different passages. SKPs in P0 and P5 were implanted into excisional wounds of nude mice in combination with freshly isolated neonatal mouse epidermal cells. Twenty days post transplantation, the wound site was photographed under a dissecting microscope and hairs generated were shown. Scale bars: 50 μm.

We first examined the rate of SKP division during culture expansion by determining the percentages of Ki67-postive cells. As shown in Fig. 1A,B, the percentage of cells expressing Ki67 decreased significantly in passage (P) 3 cells, compared to freshly isolated SKPs (P0), indicating a decrease in SKP proliferation. Then we performed SA-β-gal stain to examine cell senescence and found that the percentage of SA-β-gal-positive cells increased markedly to over 30% in P3 SKPs, compared to P0 SKPs (Fig. 1C,D), indicating that SKPs were undergoing senescence with successive culture. Next we examined alkaline phosphatase (AP) activity in SKPs, which were found largely parallel to the hair-induction activity of DP cells^8^ and SKPs^11^. The results showed that the activity of AP in SKPs decreased progressively with culture passaging, by 70% in P3 cells, compared to that in P0 SKPs (Fig. 1E,F). Finally, we tested the hair-induction activity of culture expanded SKPs by hair follicle reconstitution assay. When SKPs in P0 and P5 were implanted in combination with freshly isolated neonatal mouse epidermal cells into excisional wounds in nude mice, P5 SKPs did not induce hair genesis, while P0 SKPs did (Fig. 1G), consistent with our earlier findings^11^.

### TSA alleviates SKP senescence

**Figure 2:**
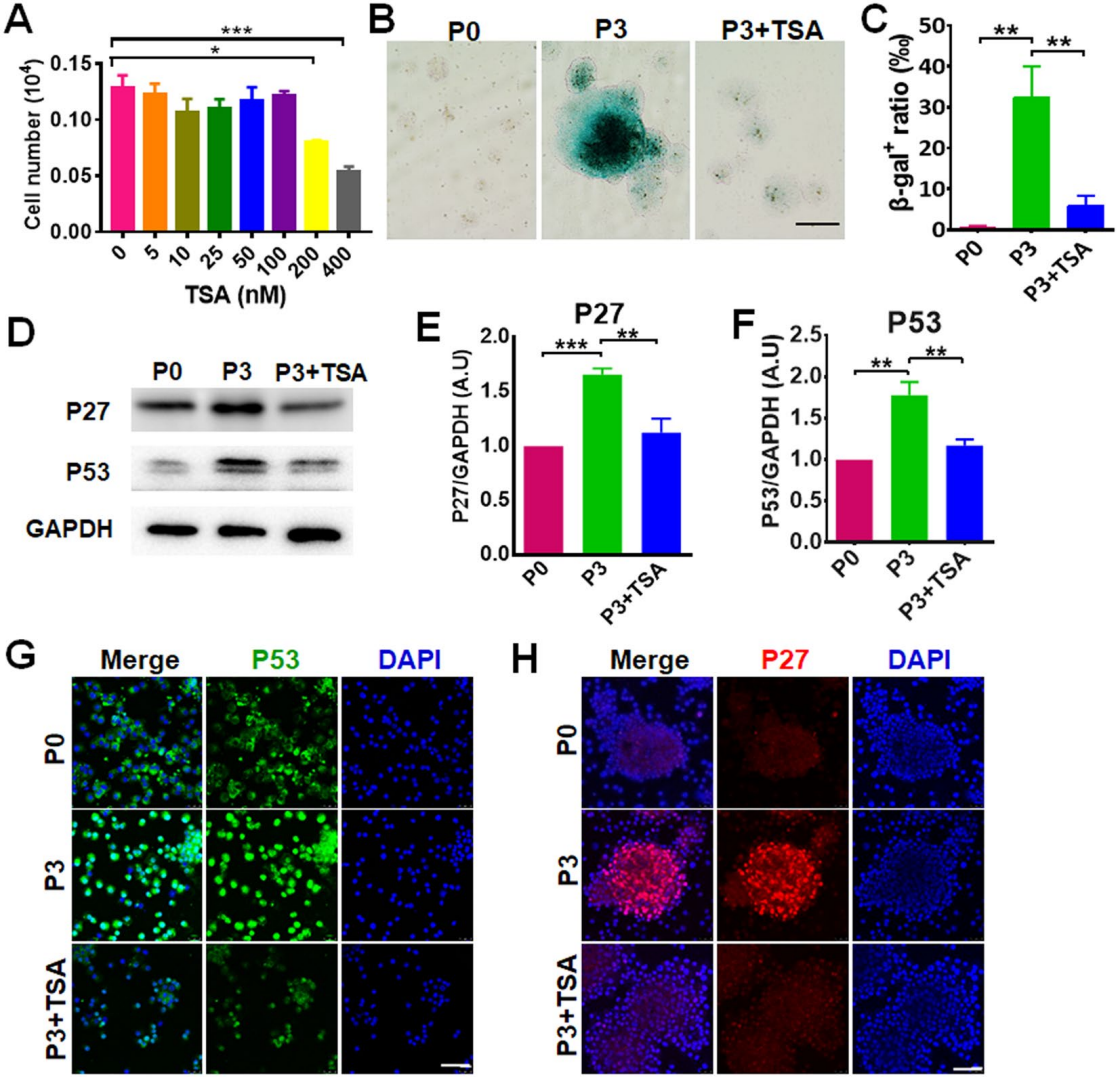
TSA alleviates SKP senescence. (**A**) SKPs in culture were treated with TSA at concentrations of 0, 5, 25, 50, 100, 200 and 400 nM for 24 h and cell numbers were counted (mean ± SEM; n = 3; *P < 0.05; ***P < 0.001). (**B**,**C**) SKPs in passage (P)0 or P3 treated without or with 100 nM TSA were stained for SA-β-galactosidase (green) and a representative image for each group was shown (**B**, Scale bars: 100 μm). The percentages of β-gal-positive SKPs were quantified using Image J (**C**). Data are represented as a mean ± SEM (n = 3; **P < 0.01). (**D**–**F**) The cells were analyzed by Western blotting with antibodies recognizing P53, P27 and GAPDH (as a loading control), respectively (**D**), and the intensity of bands of P27 (**E**) and P53 (**F**) was quantified by densitometry and normalized to GAPDH (mean ± SEM; n = 3; **P < 0.01; ***P < 0.001). (**G**,**H**) SKPs in P0 and P3 treated with or without 100 nM TSA were analyzed by immunofluorescence staining with anti-P53 (**G**) or anti-P27 (**H**) antibody. Nuclei were stained with DAPI. Scale bars: 50 μm.

In our previous studies with mesenchymal stem cells, we found that the loss of primitive cell properties were associated with deacetylation of genes involved with stem cell potency^18^. Here we examined whether TSA could restore the primitive properties of SKPs. First, we evaluated the cytotoxicity of TSA on SKPs. SKPs in P5 were treated with different concentrations of TSA (0–400 nM) for 24 h. At concentrations ≤100 nM, the growth of SKPs was unaffected; but at concentrations of 200 nM and 400 nM, the number of AKPs decreased, suggestive of toxicity of TSA to SKPs at high concentrations (Fig. 2A). Then we investigated the effect of TSA on cellular senescence of SKPs. As expected, TSA increased the acetylation level of histone H3 (Fig. S1). TSA treatment of P3 SKPs markedly decreased the number of SA-β-gal-positive cells (Fig. 2B,C). Next we examined the expression of key senescence regulators P53 and P27 by Western blot. As shown in Fig. 2D–F, the expression levels of p27 and p53 increased in P3 SKPs compared to P0 SKPs, and TSA treatment significantly down-regulated their expression. Similarly, immunofluorescence analysis of the cells confirmed the expressional changes in p53 and p27 (Fig. 2G,H).

### TSA increases AP expression in SKPs

**Figure 3:**
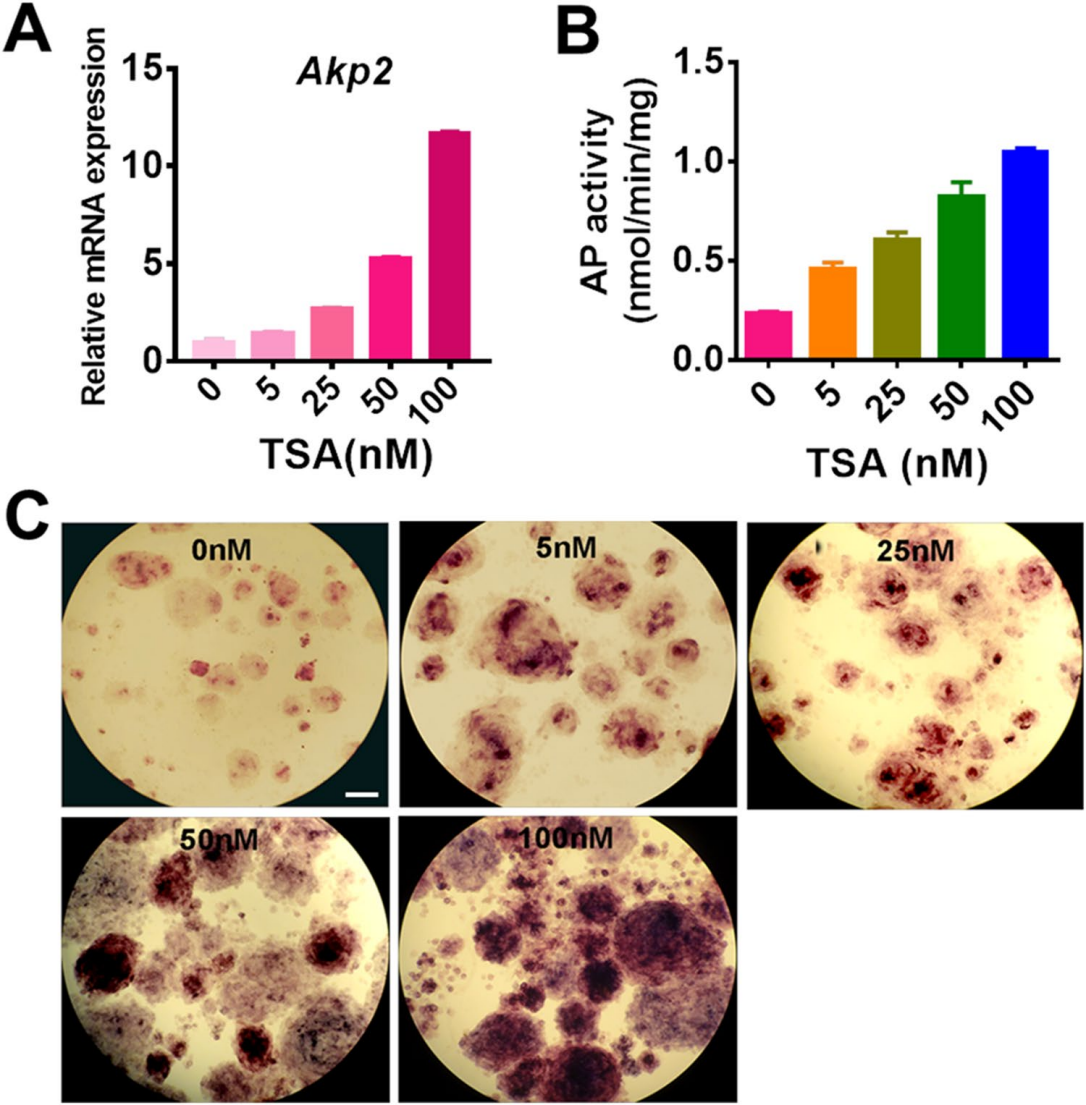
TSA increases AP expression and activity in SKPs. (**A**) The mRNA levels of *Akp2* in passage 5 SKPs treated with TSA at the concentration of 0, 5, 25, 50 and 100 nM for 24 h were analyzed by RT-PCR. Bars represent means ± SEM; technical replicates (n = 3) from one representative experiment are shown. ***P < 0.001. (**B**,**C**) AP activity of SKPs treated with TSA at the concentration of 0, 5, 25, 50, and 100 nM for 24 h was determined using an AP kit (**B**). SKPs treated with different concentrations of TSA were stained for AP activity and representative images were shown (**C**). Scale bars: 50 μm.

Real-time PCR analysis of SKPs treated with 0–100 nM TSA showed that TSA increased the expression level of *Akp2* (alkaline phosphatase gene) in a dose dependent manner (Fig. 3A). In consistence, TSA treatment improved the AP activity of SKPs with increasing concentrations and 100 nM TSA induced the highest AP activity (Fig. 3B,C).

### TSA restores the hair follicle-inductive capacity of SKPs

**Figure 4:**
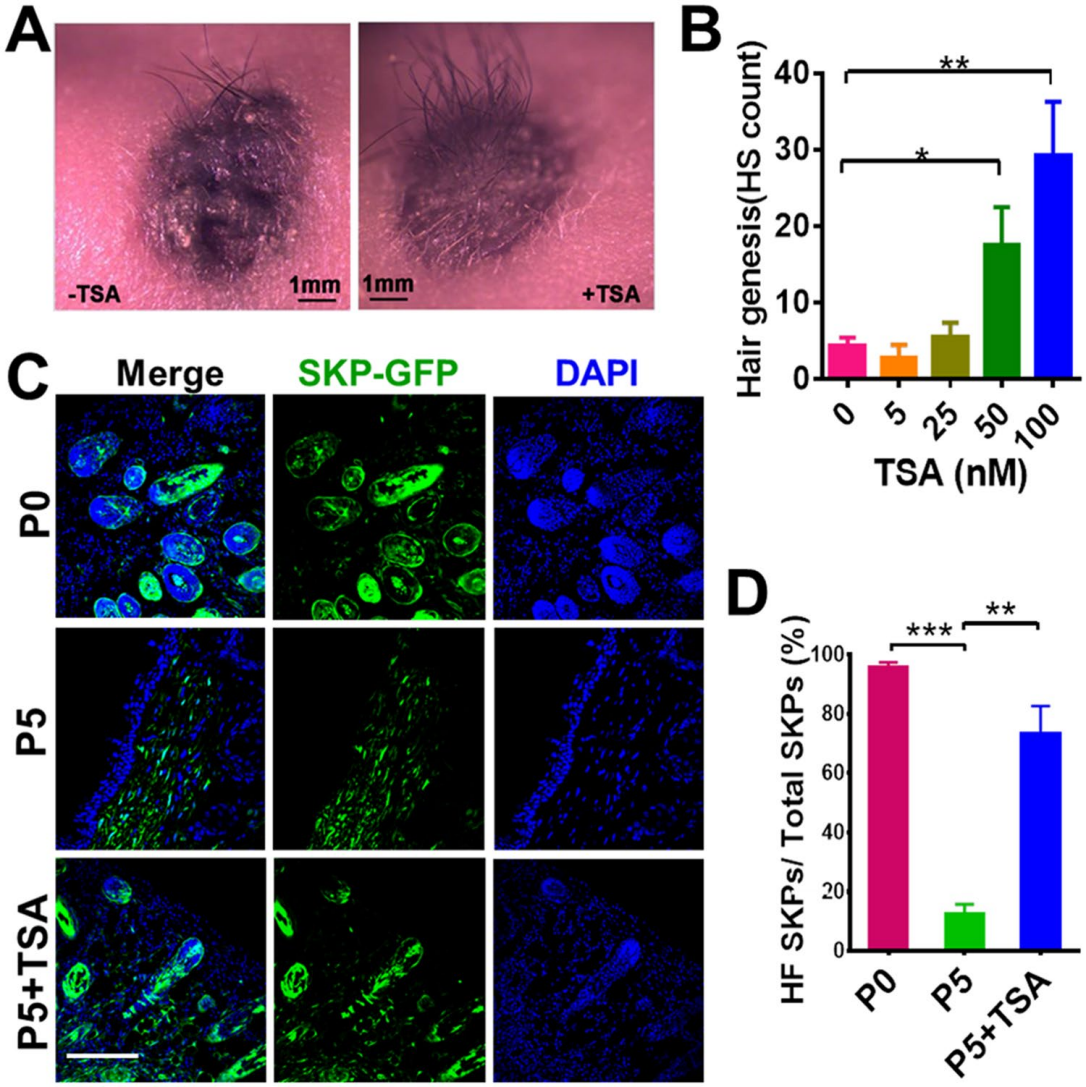
TSA improves the hair follicle-inductive capacity of culture expanded SKPs. (**A**,**B**) Hair genesis of SKPs treated with different concentration of TSA. SKPs in P5 were treated with different concentrations of TSA and implanted into excisional wounds in nude mice in combination with freshly isolated neonatal mouse epidermal cells. Twenty-one days post transplantation, hairs generated at the wound sites were photographed (**A**), and the number of hairs in each wound was counted (**B**, mean ± SEM; n = 5; *P < 0.05; ** *P* < 0.01). Scale bars: 1 mm. (**C**,**D**) Immunofluorescence staining of tissue sections of the wounds with DAPI and anti-GFP antibody showed that more neogenic hair follicles containing GFP-expressing SKPs were found in wounds receiving TSA-treated SKPs, compared to wounds receiving SKPs without TSA treatment (**C**). Error Bars represent means ± SEM; n = 5; **P < 0.01; ***P < 0.001. Scale bars: 100 μm.

Next, we examined whether supplementation of TSA to the culture of SKPs could restore their hair-inductive capacity. Hair follicle reconstitution assay (in combination with freshly isolated neonatal murine epidermal cells) showed that treatment of P5 SKPs with TSA (0–100 nM) for 24 h significantly increased their ability in inducing hair neogenesis in nude mice in a dose-dependent manner (Fig. 4A,B). Immunofluorescence staining for GPF-expressing SKPs showed that the higher presence of SKPs in the neogenic skin in mice receiving TSA (at 100 nM)-treated SKPs compared to SKPs without treatment (Fig. 4C,D).

### TSA increases the expression and activity of BMPs in SKPs

**Figure 5:**
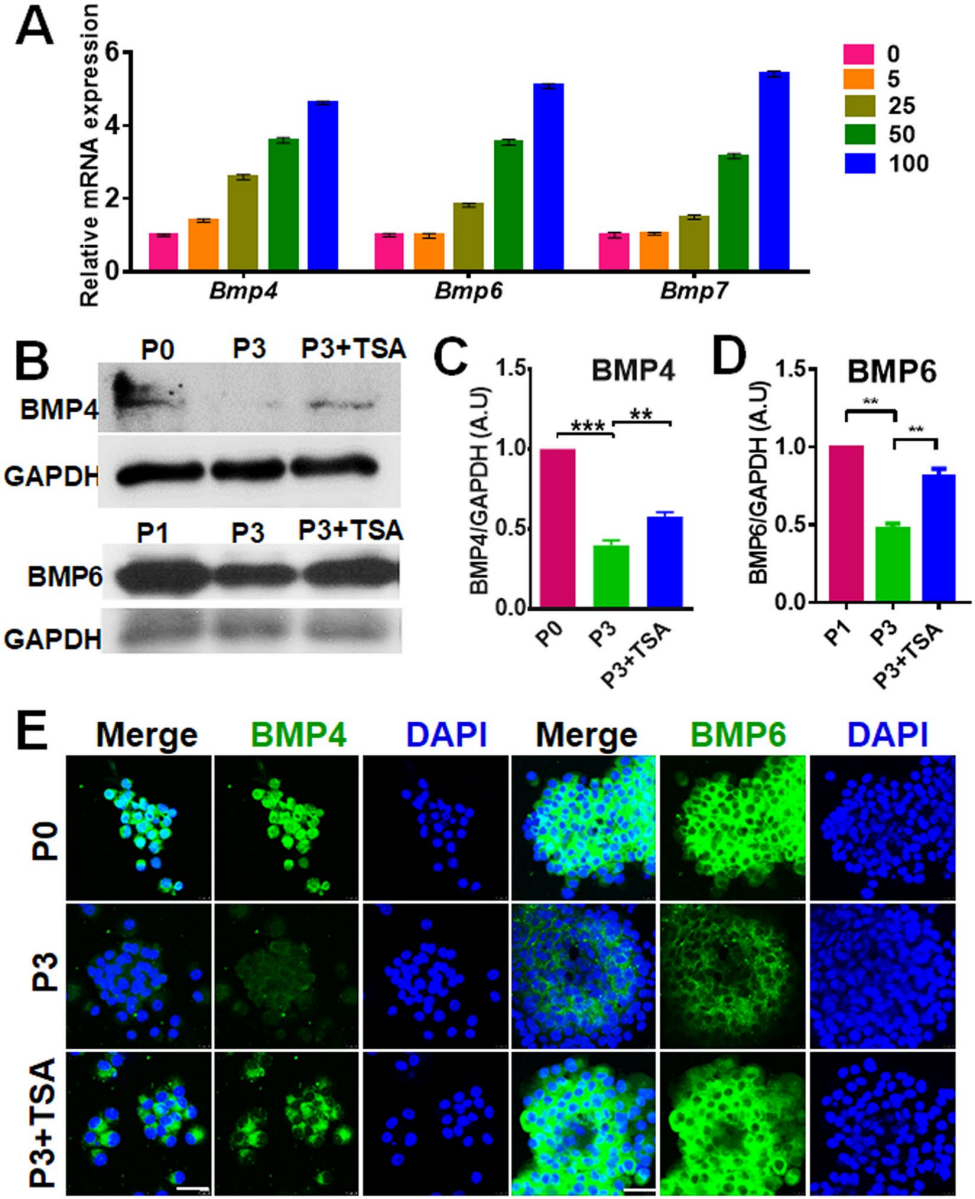
TSA rescues BMP expression in SKPs. (**A**) Passage 3 SKPs were treated with TSA at the concentration of 0, 5, 25, 50 and100 nM for 24 h and the mRNA levels of *Bmp4*, *Bmp6* and *Bmp7* in the cells were analyzed by RT-PCR. Bars represent means ± SEM; technical replicates (n = 3) from one representative experiment are shown. ***P < 0.001. (**B**–**D**) The lysate of the cells were analyzed by Western blotting for the protein expression levels of BMP4 and BMP6, and the intensity of the bands was measured by densitometry and normalized to GAPDH (**C**,**D**). mean ± SEM; **P < 0.01; ***P < 0.001. (**E**) P0 and P3 SKPs treated with or without 100 nM TSA were analyzed by immunofluorescence staining for the expression of BMP4 and BMP6. Nuclei were stained with DAPI. Scale bars: 25 μm.

Our previous studies have shown that BMPs (a combination of BMP2, BMP4, and BMP6), but not Wnts (a combination of Wnt3a, Wnt5a, and Wnt10b), significantly increased the hair inductive ability of SKPs associated with increased AP activity^11^. Here we examined the influence of TSA on BMP signaling in SKPs. First we analyzed the effect of TSA on the expression of BMPs. Treatment of SKPs in P5 with TSA (0–100 nM) for 24 h increased the mRNA levels of *BMP4*, *BMP6* and *BMP7* (Fig. 5A). Similarly, Western blot analysis indicated that treatment of the cells with 100 nM TSA increased the protein levels of BMP4 and BMP6 (Fig. 5B–D). Immunofluorescence analysis of SKPs confirmed the upregulation of the proteins after TSA treatment (Fig. 5E). As expected, levels of H3K9/K14ac in the promoter regions of *BMP2*, *BMP4* and *BMP6* were increased in P3/P5 TSA-treated SKPs (Fig. S2).

**Figure 6:**
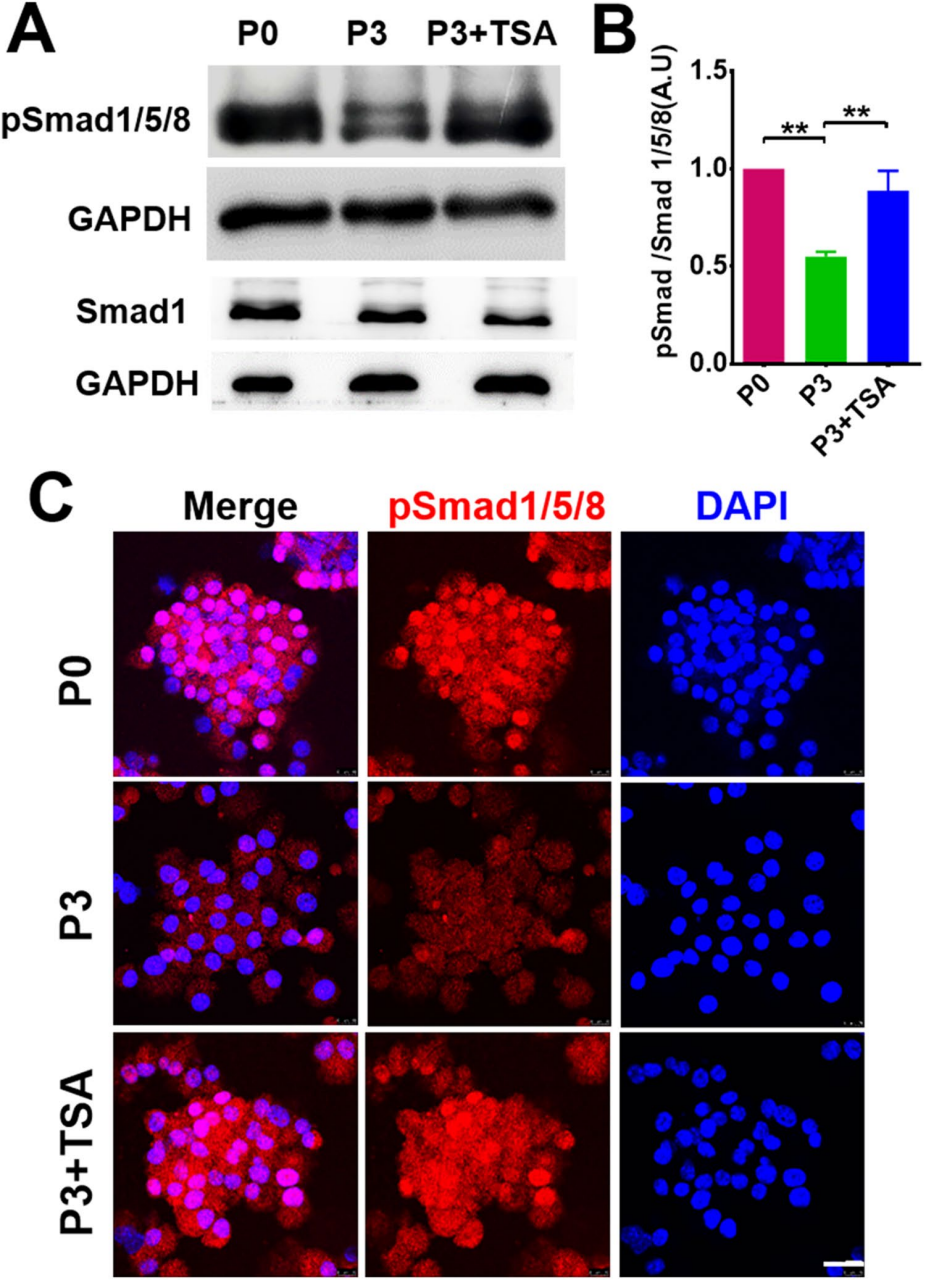
TSA enhanced BMP signaling in SKPs. (**A**–**C**) SKPs in passage (P)0 and P3 treated with or without TSA (100 nM) were analyzed by Western blotting for the expression of Smad1 and phosphorylated (p) Samd1/5/8 (**A**). The intensity of bands was quantified by densitometry (**B**). mean ± SEM; **P < 0.01. (**C**) The cells were also analyzed by immunofluorescence staining for the expression of pSamd1/5/8. Nuclei were stained with DAPI. Scale bars: 25 μm.

In canonical BMP pathway, binding of BMPs to their receptors induce a downstream cascade leading to the phosphorylation of Smads. We determined the levels of phosphorylated Smad1/5/8 (pSmad1/5/8) in SKPs after TSA treatment by Western blot. As illustrated in Fig. 6A,B, TSA treatment of SKPs in P3 significantly increased the phosphorylation level of Smad1/5/8 to a level comparative to that in P0 SKPs. Immunofluorescence analysis of the cells showed that TSA treatment increased the accumulation of pSmad1/5/8 in P3 SKPs, particularly in the nuclei (Fig. 6C).

## Discussion

SKPs have been shown to migrate into existing DPs of the hair follicle when being intracutaneously injected and form *de novo* DPs when implanted in combination with neonatal epidermal cells into excisional wounds^10,11^, suggesting their therapeutic potential in hair regeneration. However, their hair forming capacity decreases progressively during culture expansion, in conventional static suspension culture condition^11^, and in stirred suspension bioreactors^12^. To maintain or recover the hair follicle inductive capacity of SKPs after culture expansion is a pre-requirement for the development of SKPs based therapies and tissue engineering. In this study, we found that supplementation of TSA into the culture of SKPs could largely restore the hair follicle forming capacity of the cells.

The epigenetic instability is an important cause for the loss of key properties of adult stem cells after culture. In our previous study, we found that the levels of histone H3 acetylation in K9 and K14 were closely associated with the differentiation potential of human mesenchymal stem cells; with successive passaging in culture, human mesenchymal stem cells showed reduced multipotent differentiation capacity accompanied by decreased levels of histone H3 acetylation in K9 and K14, and supplementation of basic fibroblast growth factor could partially restored the acetylation levels in these sites and the differentiation potency of the cells^18,19^. So, in the present study, we focused on the alteration of the level of histone H3 acetylation in K9 and K14 in SKPs after TSA treatment, and found it relevant to the hair follicle inductive ability of the cells, despite that TSA may potentially increase the acetylation of other sites in histone/non-histone proteins.

TSA and other HDAC inhibitors such as valproic acid (VPA) have been shown to greatly improve the reprogramming efficiency of somatic cells to induced pluripotent stem cells^20^. In DP cell culture, expansion of the cells in adherent culture lead to a marked reduction of their hair induction capacity; 3D spheroid culture induces a partial reprogramming of the cells resulting in a partial recovery of their hair induction property^21^. In this study, we found that supplementation of TSA to culture expanded SKPs led to a remarkable recovery of their hair induction ability, which was associated with alleviation of senescence and significantly increased AP activity.

BMPs are important in orchestrating tissue architecture by inducing a group of pivotal morphogenetic signals. Embryonic inhibition of BMP signaling by conditional targeting of Bmpr1a blocks hair lineage specification and/or differentiation^22,23^. In stem cells, the presence of BMPs in culture has been shown to be crucial for the maintenance of their primitive states^24^. BMP signaling in DP cells is required for the maintenance of their hair follicle-inductive properties. In the absence of BMP signals, DPs lose their signature characteristics *in vitro* and fail to generate hair follicles when engrafted with epithelial stem cells *in vivo*^8^. In our previous study, we found that BMP2, BMP 4 or BMP6, with BMP4 in particular, could increase the AP activity of culture expanded SKPs, and supplementation of BMP4 to the culture increased the hair induction ability of SKPs^11^. In this study, we demonstrated that supplementation of TSA to SKP culture increased the histone acetylation levels of histone H3 K9 and K14 in the promotor regions of BMP2, BMP4 and BMP6, which was associated with increased expression levels of the genes. These results suggest an autocrine mechanism of BMPs in SKPs, and TSA likely, at least in part, restores the hair inductive ability of culture expanded SKPs by increasing the expression of BMPs.

## Materials and Methods

### Mice

C57BL/6 (7 weeks old) and BALB/c nu/nu mice (5 weeks old) were purchased from Guangdong Medical Laboratory Animal Center (Guangzhou, People’s Republic of China). C57BL/green fluorescent protein (GFP) mice (6 weeks old) were obtained from Cyagen Biosciences (Guangzhou, People’s Republic of China). The animals were maintained in a temperature controlled environment (20 °C ± 1 °C) with access to food and water throughout the experiment. All animal procedures were performed with the approval of the Animal Ethics Committee of Tsinghua University. All experiments were performed in accordance with relevant guidelines and regulations.

### Isolation and culture of SKPs

Murine SKPs were isolated as we previously described^11^. Briefly, we generated SKPs from neonatal mouse dorsal skin of C57BL/6 or C57BL/GFP mice within 1~3 days after birth. Dissected skin tissue was minced into 2–3 mm^2^ size pieces using a sterile razor blade, and then transferred into a 15 ml conical tube for digestion with 0.3% Dispase II (sigma) for 90 min at 37 °C. The epidermis was manually removed from the tissue. The dermis was digested with 0.2% collagenase I for 30~40 min at 37 °C with shaking, then filtered through a 40 µm cell strainer. The dissociated cells were plated in a 10 cm non-treated dish using 10 ml Dulbecco’s modified Eagle’s medium (DMEM)/F12, 3:1 (Gibco) containing B27 (Gibco), 20 ng/ml epidermal growth factor (EGF, Peprotech) and 40 ng/ml basal fibroblast growth factor (bFGF, Peprotech) and incubated in a 37 °C, 5% CO_2_ tissue culture incubator.

### Hair follicle regeneration assay

BALB/c nu/nu mice (5–6 weeks old) were anesthetized with an intraperitoneal (i.p.) injection of 50 mg/kg sodium pentobarbital. Two symmetrical 3-mm-diameter circular wounds with full thickness skin were created on the back using a skin biopsy punch as previously described^11^. 2 × 10^6^ SKPs were mixed with 1 × 10^6^ neonatal mouse epidermal cells in 10 μl Matrigel (BD Biosciences). The cells-Matrigel was implanted into the excisional wound, covered with Tegaderm (3 M) transparent dressing, and further covered with self-adhering elastic bandage. Three to four weeks later, mice were sacrificed for counting hair number and histological analysis.

### SA-β-gal staining

SA-β-gal staining was carried out using a SA-β-gal staining kit (Beyotime Biotechnology, China). SKP spheres were cytospun onto slides and fixed with 4% paraformaldehyde for 30 min. Samples were washed with PBS, stained with SA-β-gal staining solution at 37 °C overnight and visualized under a light microscope.

### Alkaline phosphatase activity and AP staining

AP activity of SKPs was measured as previously described^11^. Briefly, for quantitative AP measurements cultured SKPs were lysed using a buffer containing 0.1% Triton X-100 (Beyotime, China). The cell supernatant was collected into a 96-well plate. Next, substrates and p-nitrophenol from Alkaline Phosphatase Assay Kit (Beyotime, China) were subsequently added and incubated for 10 min at 37  °C. Finally, the AP activity was determined at the wavelength of 405 nm. For AP staining, cultured SKPs were stained with AP Staining Kit (Beyotime Biotechnology, China) following the manufacturer’s protocol. Briefly, cells were washed twice with PBS, and fixed with 4% paraformaldehyde for 30 min at room temperature. Staining buffer was then added to the cells. After incubation at 37 °C for 2 h, the samples were observed under a light microscope.

### Cell proliferation assay

SKP proliferation was evaluated using cell-counting kit-8 (CCK-8). SKPs were seeded in 96-well plates (5,000 cells per well) and incubated for 48 h. The culture was then supplemented with different concentrations of TSA (Sigma, USA) and incubated for another 24 hours at 37 °C in 5% CO_2_. After treatment with 10 µl CCK-8 for another 3 h, the culture was then subjected to spectrophotometric analysis with a microplate reader (BioTek) with absorbance at 450 nm. Cells cultured in the absence of CCK-8 and culture medium alone were used as controls.

### Real-Time PCR analysis

Total RNA was extracted using TRIzol (Invitrogen, USA) according to the manufacturer’s instructions. Reverse transcription (RT) was performed using Superscript II reverse transcriptase (Invitrogen, USA). Real-time PCR was performed in a 20 μl reaction volume using SYBR Green Real-Time PCR Master Mix (Toyobo, Japan) on an ABI 7500 QPCR System. The relative expression values of each gene were determined by comparing to glyceraldehyde- 3-phosphate dehydrogenase (GAPDH) expression level in each sample. Normalization and fold changes were calculated using the − ΔΔCt method. Primer sets are shown in Tables 1 and 2.

**Table 1:** Primers used for Real-time PCR.

Gene	Sense	Antisense
*Akp2*	TCGGAACAACCTGACTGACCC	CTGCTTGGCCTTACCCTCATG
*Bmp4*	CAGGGAACCGGGCTTGAG	CTGGGATGCTGCTGAGGTTG
*Bmp6*	TGTGGTGACTCGGGATGGAC	ATGAAGGGCTGCTTGTCGTAAG
*Bmp7*	ACCCTCGATACCACCATCGG	GCTCCCGGATGTAGTCCTT

**Table 2:** Primers used for Real-time PCR for ChIP.

Gene	Sense (from 5′-3′)	Antisense (from 5′-3′)
*Bmp2*	CCGACGACAGCAGCAGCCTT	AAGACTGGATCCGCCGGGCG
*Bmp4*	GCCATTCCGTAGTGCCATTC	CATGATTCTTGGGAGCCAATC
*Bmp6*	TGTGGTGACTCGGGATGGAC	ATGAAGGGCTGCTTGTCGTAAG
*GAPDH*	ACTGAGCAAGAGAGGCCCTA	TATGGGGGTCTGGGATGGAA
Intergenic region	TGGGCATATCCCTGGAGCTT	GGCCATCCCACAGTCACAAC

### Immunofluorescence staining

Skin tissues embedded in OCT were sectioned in 10 µm thickness. SKP spheres were cytospun onto slides and fixed with 4% paraformaldehyde (Sigma) for 30 min at room temperature. Samples were washed with PBS and blocked with 3% BSA/PBS containing 0.2% Triton-X 100 (Sigma) at 37 °C for 1 h, and then incubated with primary antibodies in 1% BSA/PBS at appropriate concentrations, respectively, at 4 °C overnight: Trp53 (rabbit, 1:200, Bioworld, BS1278), p27 (mouse, 1:200, BD Bioscience, #610242), phospho-Smad1,5,8 (rabbit, 1:100, Cell signaling technology, #13820), BMP-4 (rabbit, 1:100, GeneTex, GTX100874), BMP-6 (rabbit, 1:100, Abcam, ab155963), Samples were then stained with FITC-conjugated secondary antibodies (Alexa Flours, Invitrogen). Nuclei were stained with 4′,6-diamidino-2-phenylindole (DAPI). Samples were examined under confocal laser scanning microscope (FV1000, Olympus, Japan).

### Western blotting

Western blotting was performed as previously described^25,26^. For preparation of total cell lysates, cells were lysed in 1% SDS lysis buffer (25 mMTris-HCl (Ph 6.8), 50 mM DTT, 8% glycerin, 2.5% sucrose). Equal amounts (10 μg/lane) of total cell protein were separated on 10% polyacrylamide gel and transferred onto a nitrocellulose membrane. Membranes were blocked for 1 hours at room temperature in Tris-buffered saline (50 mM Tris-HCl, 150 mM NaCl, pH 7.4) containing 0.1% Tween 20 and 5% non-fat powdered milk, followed by overnight incubation at 4 °C with GAPDH (mouse, ZSGB-BIO, 1:3000, TA-08), Trp53 (rabbit, 1:1000, Bioworld, BS1278), p27 (mouse, 1:2000, BD Bioscience, #610242), phospho-Smad1,5,8 (rabbit, 1:1000, Cell signaling technology, #13820), BMP-4 (rabbit, 1:1000, GeneTex, GTX100874), BMP-6 (rabbit, 1:1000, Abcam, ab155963), Smad-1 (rabbit, 1:1000, cell signaling technology, #6944). After washing and incubation with appropriate HRP-conjugated secondary anti-rabbit or mouse IgG antibodies (Jackson ImmunoResearch, #711-005-152 or #715-005-151), blots were developed using an enhanced chemiluminescence kit (ECL Kit, Bio-Rad) and then exposed to x-ray film (Fuji film, # super RX-N-C). The images were scanned using an imaging scanning system (EPSON Scan; L365). Quantification of densitometry was performed using Image J.

### Statistical analysis

All values are expressed as mean ± SEM. One-way ANOVA was used for multiple group comparisons. A probability (*P*) value < 0.05 was considered significant.

## Supplementary information

Supplemental Material
