# Supplementary material for: TSA restores hair follicle-inductive capacity of skin-derived precursors
Source: Sci Rep. 2019 Feb 27;9:2867. doi: 10.1038/s41598-019-39394-w (PMC6393485; doi:10.1038/s41598-019-39394-w)
Supplement: Supplementary file 1 — Supplemental Material [file 41598_2019_39394_MOESM1_ESM.pdf]

Supplemental Material

TSA restores hair follicle-inductive capacity of skin-derived precursors

Ling Guo[1,3*], Xiaoxiao Wang[1,2,3*] Jifan Yuan[3*], Meishu Zhu[4], Xiaobing Fu[5], Ren-He Xu[6], Chuanyue Wu[7#], Yaojiong Wu[1,2#]

[1]State Key Laboratory of Chemical Oncogenomics, and the the Shenzhen Key Laboratory of Health Sciences and Technology, Graduate School at Shenzhen, Tsinghua University, China

[2]Tsinghua-Berkeley Shenzhen Institute (TBSI), Tsinghua University, China

[3]Guangdong Provincial Key Laboratory of Cell Microenvironment and Disease Research， Shenzhen Key Laboratory of Cell Microenvironment, and Department of Biology, Academy for Advanced Interdisciplinary Studies, Southern University of Science and Technology, China

[4]Shenzhen Second People's Hospital (The First Hospital Affiliated to Shenzhen University), Shenzhen, China.

[5]Wound Healing and Cell Biology Laboratory, Institute of Basic Medical Science, Chinese PLA General Hospital, Beijing, China; Stem Cell and Tissue Regeneration Laboratory, The First Affiliated Hospital, General Hospital of PLA, Beijing, China.

[6]University of Macau, Institute of Translational Medicine, and Centre of Reproduction, Development and Aging, Faculty of Health Sciences, Taipa, Macau, China.

[7]Department of Pathology, University of Pittsburgh School of Medicine, Pittsburgh, PA 15261, USA

*These authors contributed equally to this work

#Correspondence should be addressed to Y.W. (wu.yaojiong@sz.tsinghua.edu.cn) or C.W.

(wucy@sustc.edu.cn)

Correspondence: Yaojiong Wu, MD, PhD, L406A, Tsinghua Campus, The University Town, Shenzhen, China. Tel/Fax: 755-2603-6348. Email: wu.yaojiong@sz.tsinghua.edu.cn

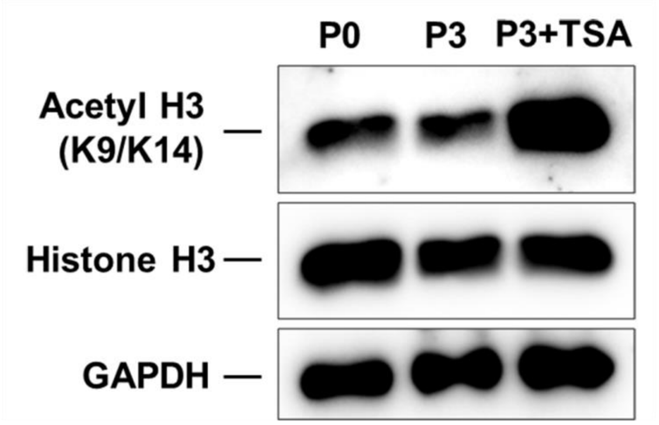

Supplementary Fig. 1 TSA increased the acetylation level of histone H3. P0 and P3 SKPs treated with or without 100 nM TSA for 24 h were analyzed by Western blotting for the protein expression levels of Histone H3 and Acetyl H3(K9/K14), and GAPDH.

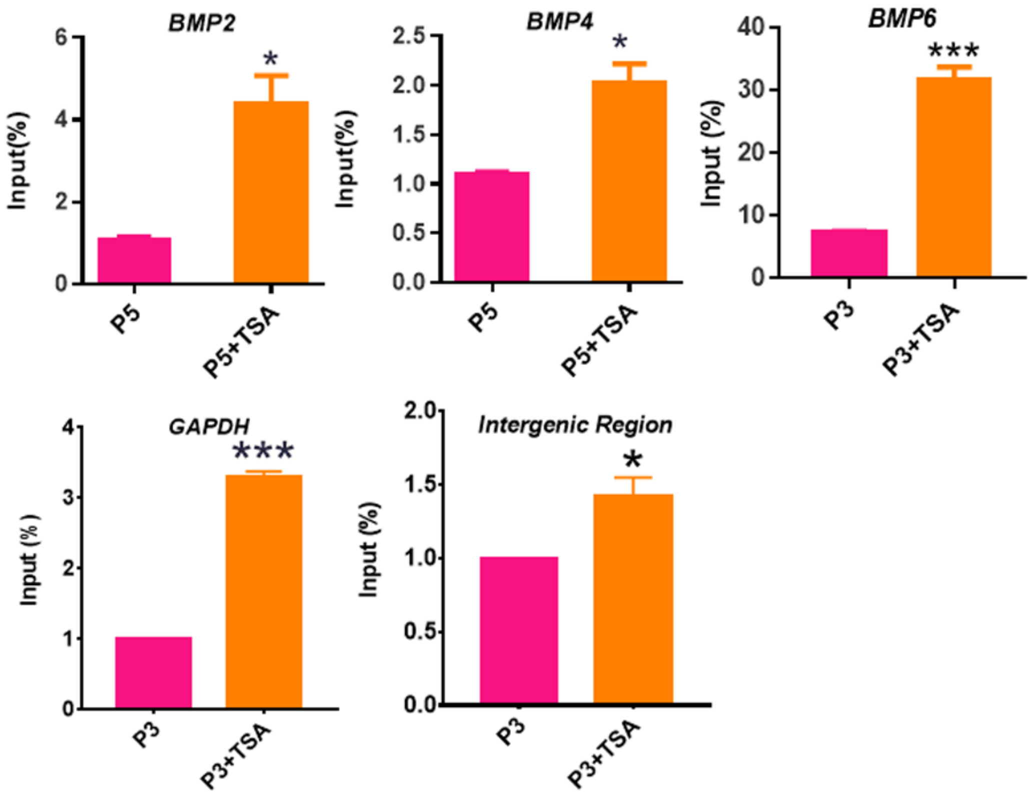

Supplementary Fig. 2 Histone acetylation alterations in TSA treated SKPs. ChIP analysis of the levels of acylation of histone H3 at K9/14 in the promoter regions of *BMP2, BMP4, BMP6 and GAPDH* and in the intergenic region in of SKPs in passage (P) 3 or 5 treated with or without 100 nM TSA for 24 h.
